# Living in a Disadvantaged Neighborhood Affects Neural Processing of Facial Trustworthiness

**DOI:** 10.3389/fpsyg.2020.00409

**Published:** 2020-03-26

**Authors:** Shou-An A. Chang, Arielle Baskin-Sommers

**Affiliations:** Department of Psychology, Yale University, New Haven, CT, United States

**Keywords:** neighborhood disorder, facial trustworthiness, late positive potential, crime, trust

## Abstract

Neighborhood disorder (i.e., physical or social decay) is associated with decreased trust, which reinforces criminal behavior among some individuals in these communities. However, past research largely is descriptive and has not measured processes underlying trust. Using behavioral and neural indices [the late positive potential (LPP), a marker of salience elaboration] in a sample of adults (*N* = 55), we examined the association between perceived neighborhood disorder and facial trustworthiness perception as well as the potential moderating role of trustworthiness perception on the association between PND and criminal behavior. Individuals with higher perceived neighborhood disorder displayed less LPP differentiation between untrustworthy and trustworthy faces. Moreover, individuals with higher perceived neighborhood disorder and less LPP differentiation were less likely to commit a variety of crimes, whereas those with higher perceptions of neighborhood disorder and high LPP differentiation were more likely to commit a variety of crimes. Combined, these findings suggest that similarly processing trustworthy and untrustworthy faces, as indexed by less LPP differentiation, may reflect an adaptation among those with higher perceived neighborhood disorder that mitigates against deviant behavior and contacts with the law. Understanding the intersection between neighborhood characteristics and individual-level cognitive-affective processing may provide insight into what shapes beliefs and behaviors about important social information.

## Introduction

Since the 2000’s, the number of distressed neighborhoods in the United States grew by nearly three-quarters (Kneebone, 2014). These neighborhoods are typically defined as having at least 40% of its residents living in poverty and are associated with an array of challenges that negatively impact its residents – whether they are poor or not. For example, distressed neighborhoods are high in neighborhood disorder, which refers to observed or perceived physical (e.g., graffiti, litter) and social (e.g., prostitution, gang activity) decay. Residents in neighborhoods with high disorder exhibit poorer physical and mental health and are at higher risk for being both the victims and perpetrators of crime (see Freedman and Woods, 2013 for review). Researchers suggest that the elevated rates of social problems (e.g., crime) in neighborhoods with high levels of disorder may relate to weakened trust in one’s neighbor and in individuals outside of the neighborhood (Sampson et al., 1997, 2008; Freedman and Woods, 2013). Notably, residents in neighborhoods marked by high disorder report increased fear of victimization by others (Ross and Mirowsky, 2009), decreased trust in their neighbors and in general (Ross et al., 2001; Marschall and Stolle, 2004; Ross, 2011), and reduced trust in the police (Nix et al., 2014). Despite this research showing that high neighborhood disorder is associated with the erosion of trust (Ross et al., 2001; Ross, 2011; Steenbeek and Hipp, 2011), no research has examined the social cognitive process that underlie the formation and maintenance of trust.

Individuals form stable trustworthiness judgments about another person based solely on their facial features, even in the absence of information about the other person’s behavior or emotional state (Oosterhof and Todorov, 2008). These judgments of trustworthiness are formed within just 100 ms of exposure to the face (Willis and Todorov, 2006). Furthermore, they affect the ways in which individuals decide to engage with others (e.g., investing more money in trustworthy vs. untrustworthy faces; Chang et al., 2010; Ewing et al., 2015; approaching trustworthy faces faster than untrustworthy faces; Slepian et al., 2017). Thus, facial trustworthiness perception is an important social cue that can influence and guide behavior.

Considering how quickly judgments of trustworthiness are formed, the high temporal resolution of event-related potentials (ERPs) makes them particularly well-suited for investigating the neural processing that underlies these rapid judgments. ERPs can reveal information about the specific cognitive processes (e.g., face vs. emotion processing) that relate to trustworthiness judgments. Past research indicates that facial trustworthiness elicits distinct patterns of neural activity. Untrustworthy faces evoke larger brain responses by 300 ms [i.e., referred to as the late positive potential (LPP), a measure of salience elaboration; Foti et al., 2009; Hajcak et al., 2009]. Compared to subjectively (Marzi et al., 2014; Lischke et al., 2017) and pre-classified (Yang et al., 2011) neutral and trustworthy faces, untrustworthy faces evoke larger LPPs. Taken together, these studies suggest that untrustworthy faces are more salient and recruit more neural resources to process than trustworthy and neutral faces in general.

However, the majority of research on trustworthiness perception has been conducted in undergraduate samples (e.g., Engell et al., 2007; Todorov et al., 2008; Oosterhof and Todorov, 2009; Todorov et al., 2009; Dotsch and Todorov, 2012) or in lesion patients (Adolphs et al., 1998; Todorov and Duchaine, 2008; Todorov and Olson, 2008). No studies have examined how a social context, like neighborhood disorder, differentially impacts processes underlying facial trustworthiness perception and connects to criminal behavior. Exploring the links between neighborhood disorder and trustworthiness processing are especially important given the two separate lines of research showing that trust behavior may be affected by the experience of neighborhood disorder and that trustworthiness perception is essential for promoting social engagement.

The goal of the present study was to investigate the association between perceived neighborhood disorder (PND) and processing of facial trustworthiness. We opted to use a measure of perceived neighborhood disorder, as opposed to census data, because it assessed one’s recognition of visible cues in their neighborhood that indicate lack of order and social control (Ross and Mirowsky, 1999; Matthews et al., 2019). This assessment of self-reported perceptions of these neighborhood cues was theoretically aligned with the concepts related to perception of facial cues that might signal trustworthiness. First, we explored the association between PND and processing (i.e., ratings and LPP) of facial trustworthiness. Second, given that not all individuals in neighborhoods marked by disorder engage in criminal behavior, we explored whether the association between PND and processes underlying trustworthiness perception predicted engagement in criminal behavior.

## Materials and Methods

### Participants

Participants were recruited from New Haven County, Connecticut. New Haven ranks in the 95th percentile for crime (Note: Data accessed from https://www.neighborhoodscout.com/ct/new-haven/crime on 9/15/19). Additionally, the County contains blocks of areas ranked nationally in the 100th percentile of neighborhood disadvantage, as measured by the Area Deprivation Index (data accessed on 9/15/19).^1^ Adults aged 18–66 (*M* = 41, *SD* = 13) were recruited through flyers advertising for individuals who engage in risk-taking behavior (e.g., gambling, alcohol, and drug use). These demographic features, combined with our targeted recruitment of “risk-takers,” resulted in a sample with varied exposure to neighborhood disorder and enriched for criminal behavior (see Table 1 for sample characteristics).

**TABLE 1:** Sample characteristics (*n* = 55).

	*n*	%
**Race**		
White	17	30.91
Black	35	63.64
Asian/America Indian	2	3.63
Biracial	1	1.82
**Education**		
Some high school or below	12	21.82
High school diploma	18	32.73
Some college	14	25.45
College/Graduate degree	11	20.00
**Household income**		
<$12,000	26	47.27
12,000–$25,000	14	25.45
25,001–$50,000	9	16.36
>$50,000	6	10.91
**Committed a crime**		
Violent	21	38.18
Non-violent	37	67.27
Both	19	34.54

*A priori* power analyses, based on previous studies on related topics (e.g., neural processing of facial trustworthiness perception (Yang et al., 2011; Marzi et al., 2014); were conducted using G^∗^Power statistical software (Faul et al., 2007). Power analyses indicated that a sample size of 50 participants would result in sufficient (80%) power to detect a moderate effect size for the omnibus interaction between level of trustworthiness of the face and one continuous predictor. All measures, manipulations, and exclusions in the study are disclosed in the sections that follow.

A prescreen phone interview and initial session including an in-person clinical assessment were used to exclude individuals who were younger than 18 or over 75, had performed below a fourth-grade level of a standardized measure of reading (WRAT-III; Wilkinson, 1993), who scored below 70 on a brief measure of IQ (Shipley; Zachary, 1986), who had diagnoses of schizophrenia, bipolar disorder, or psychosis not otherwise specified, or who had a history of medical problems (e.g., uncorrectable auditory or visual deficits, seizures, head injury with loss of consciousness >30 min, color blindness) that may impact their comprehension of materials and performance on the task. Participants also completed a self-report measure of neighborhood disorder during the initial session. During the second session, participants completed the experimental task. All participants provided written informed consent and experimental procedures were approved by the Yale University Human Investigation Committee. Participants were paid $10/h per session.

### Measures

#### Perceived Neighborhood Disorder Scale (PND; Ross and Mirowsky, 1999)

The PND scale is a 15-item self-report measure of physical (i.e., graffiti, abandoned buildings) and social (i.e., police protection, loitering) order in one’s community. Scores are summated into a total PND score (range 15–60) with higher scores indicating higher levels of PND in one’s community (sample *M* = 32.62, *SD* = 9.70). The PND scale has been found to be a reliable and valid measure of PND (Ross and Mirowsky, 1999). For this sample, internal consistency (i.e., reliability) was acceptable (Cronbach’s α = 0.75).

#### Criminal Behavior

All participants were asked if they ever committed a crime, even if they were never officially charged. Participant self-report was corroborated using the State of Connecticut Department of Correction inmate database. Crimes were coded as non-violent or violent based on state legal code. A variable was created that indexed the variety of types of crimes that the participant reported (0 = none, 1 = one type; non-violent or violent, 2 = two types; non-violent and violent). This type of variable was used because a significant portion of the sample endorsed engagement in criminal activity (see Table 1), therefore, accounting for the variety in types of criminal engagement provided a more meaningful measure of variability in criminal behavior.

### Experimental Procedure

#### Apparatus

Presentation of all stimuli and measurement of behavioral responses was controlled using the Pyschtoolbox extension in MATLAB. EEG recording was controlled by a MATLAB script and Neuroscan Synamps amplifiers and acquisition software (Compumedics, North Carolina). All tasks were presented on a Ben-Q 27-inch high performance LED gaming monitor. Participants’ eyes were at a distance of 75 cm from the screen. Participants registered their responses using a button box.

#### Stimuli

Stimuli were obtained from a set of computer-generated faces manipulated on a dimension of trustworthiness developed by Todorov and Oosterhof (2011) and Oosterhof and Todorov (2008). This dimension of trustworthiness was derived by Oosterhof and Todorov (2008) using a model-based approach, which determined the four facial features most strongly driving judgments of trustworthiness. The four facial features that constitute this dimension of trustworthiness are the brow ridge (i.e., inner eyebrows), cheekbones, chins, and nose sellion. For example, more trustworthy faces are characterized by high inner eyebrows, pronounced cheekbones, wide chins, and shallow nose sellions, whereas more untrustworthy faces are characterized by the opposite directionality of these facial features. Manipulation of this dimension (i.e., four facial features) has been used to generate faces varying on levels of trustworthiness. The faces were generated by the Todorov group using FaceGen 3.1 (Singular Inversions, 2005) and were all male, bald, Caucasian, and front facing with direct gaze. The set contained 100 unique identities varied on three levels of trustworthiness, for a total of 300 faces. The three levels of trustworthiness represented less trustworthy (−3 SD), neutral (0 SD), and more trustworthy (+3 SD) versions of each facial identity. This stimulus set has been used in previous EEG studies investigating evaluation of facial trustworthiness (Yang et al., 2011; Marzi et al., 2014). Furthermore, use of this dataset allowed the ratings of trustworthiness made by our participants to be compared against consensus ratings (Oosterhof and Todorov, 2008).

#### Trustworthiness Rating Task

Participants were instructed to rate the trustworthiness of the face that appeared on the screen. It was emphasized that participants should go with their “gut reaction” when rating the face, as faces would be presented briefly. When prompted by an instruction screen, participants rated the presented face by pressing either “trustworthy,” “neutral,” or “untrustworthy” labeled buttons on a button box with the index finger of the dominant hand.

In total, participants completed 180 trials comprised of 60 randomly selected identities that varied on 3 levels of trustworthiness. The trustworthiness ratings task consisted of 6 blocks of 30 trials. To control for repetition effects, the order of the stimuli was randomized such that the same facial identity was not presented more than once in a block. Each trial consisted of a white fixation cross presented on a black background for 500 ms, followed by presentation of a target face for 750 ms, and finally an instruction screen: “Rate the face on trustworthiness.” Participants had up to 2,250 ms to respond with a button press. If the participant did not respond within that time, the task automatically advanced to the next trial. The intertrial interval was variable between 1,000 and 2,000 ms.

### Behavioral Data Reduction

To examine agreement in rating of facial trustworthiness with respect to level of trustworthiness as determined by Todorov et al.’s original ratings (Oosterhof and Todorov, 2008), a percentage of “congruent” responses was calculated by dividing the number of rated faces that matched Todorov et al.’s original ratings (Oosterhof and Todorov, 2008) by the total number of faces presented for each level of trustworthiness (i.e., 60). Thus, a percentage of congruent ratings was calculated for untrustworthy, trustworthy, and neutral faces.

### Psychophysiological Recording and Data Reduction

EEG was continuously collected at a 2,500 Hz sampling rate from 8 AG/AgCl electrodes based on the 10–20 system (Fz, Cz, Pz, CP3, CP4, Oz, O1, O2) and referenced to the left mastoid electrode. Vertical electro-oculographic (VEOG) activity was recorded in line with the pupil above and below the left eye and utilized to correct for ocular artifact. An upper forehead electrode was used for ground. Electrode impedances for all channels were kept below 10 KΩ by lightly abrading the scalp and applying gel in the sensor cups at the start of the experimental session.

Data was processed offline using the PhysBox plugin (Curtin, 2011) within the EEGLab toolbox (Delorme and Makeig, 2004) in MATLAB. Offline processing included low-pass and high-pass filtering (2nd-order, 30 Hz low-pass Butterworth filter; 0.5 Hz high pass filter), epoching (−200 to 750 ms epochs), baseline correction (200 ms), and artifact rejection (rejection of trials with voltages exceeding ±100 V).

Waveforms were averaged based on subjective trustworthiness ratings (trustworthy, neutral, or untrustworthy). Participants were excluded from analyses if they rated fewer than 10 faces for any of the three face types (e.g., only rated five faces as trustworthy), as this resulted in insufficient trials to generate a robust ERP. Thirteen participants were eliminated for this criterion. Participants were also excluded if they had fewer than 10 valid trials for any of the three face types [e.g., controlling for electrical noise and excluding trials with significant artifact (±100 V)]. This criterion eliminated four participants. Finally, three outliers on the omnibus interaction and EEG main effects were identified using Studentized residuals (with Bonferroni-corrected *p* < 0.04) and were excluded. The final sample was 55,^2^ and data collection did not continue after data analysis began.

Based on the grand average event-related potential (ERP) waveform, previous literature examining the neural correlates of trustworthiness appraisal (Marzi et al., 2014), and EEG literature indicating that the LPP is maximal over central-parietal sites (Hajcak et al., 2010), the LPP was measured as the average amplitude over CP3 and CP4 between 450 and 750 ms. In order to confirm our electrode selection and guide further analyses, a General Linear Model (GLM) with LPP amplitude as the continuous, dependent measure and scalp site (O1, O2, Oz, Cz, Fz, Pz, CP3, CP4, and Cz) as a between-subjects factor was conducted. Results indicated a main effect of scalp site *F*(1, 54) = 45.61, *p* < 0.001, η*_*p*_^2^* = 0.46, and a within-subject interaction of scalp site *F*(7, 378) = 9.31, *p* < 0.001, η*_*p*_*^2^ = 0.15. *Post-hoc* interaction contrasts revealed that there were no significant differences between CP3 and CP4, thus further analyses were conducted by averaging amplitudes for CP3 and CP4 electrodes.

## Results

### Ratings

To examine the effect of PND on ratings of facial trustworthiness, we entered the percentage of congruent ratings in a GLM with face type (neutral, trustworthy, or untrustworthy) as a within-subjects factor and PND total score as a continuous covariate. Planned Helmert contrasts of neutral vs. trustworthy and untrustworthy faces and trustworthy vs. untrustworthy faces were conducted. To protect against violations of the assumption of sphericity, Huynh-Feldt corrected *p* values were reported for all following analyses. Results showed no significant interaction between PND score and face type *F*(1.41, 74.68) = 0.23, *p* = 0.718, η*_*p*_*^2^ = 0.004 on the percentage of congruent ratings of trustworthiness. Additionally, there was no main effect of PND on face type, *F*(1, 53) = 0.18, *p* = 0.671, η*_*p*_*^2^ = 0.003. This suggests that PND does not affect the ability to rate perceptions of facial trustworthiness in a manner congruent with established norms.

### Neural Reactivity to Faces

**FIGURE 1:**
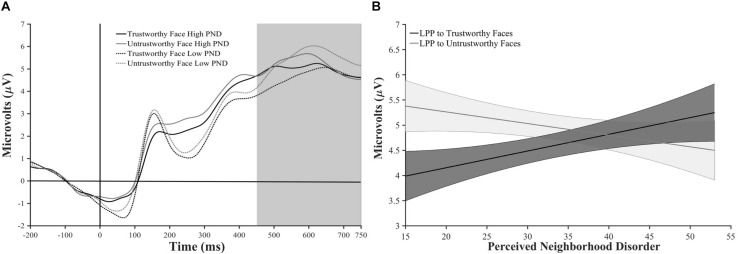
**(A)** Average ERP waveform at electrode CP4, where LPP mean amplitude was maximal for individuals low and high on PND total score. Primary analyses were conducted using continuous PND scores, and “low PND” (individuals who scored below the mean on the PND variable) and “high PND” (individuals who scored above the mean on the PND variable) groups were created solely for ease of visual representation of results. Dashed lines represent the low PND group, and the solid lines represent the high PND group. The black lines represent trials where the face was rated as trustworthy, and the gray lines represent trials where the face was rated as untrustworthy. The gray box indicates the timeframe (450–740 ms) used to derive the LPP mean amplitude measure. **(B)** Regression lines represent LPP amplitude to trustworthy and untrustworthy faces as a function of perceived neighborhood disorder (PND total; lines represent scores at ±1 SD from the mean). Error bands indicate 1 SE.

To examine the effect of PND on LPP, mean LPP amplitude was entered in a GLM with face type (neutral, trustworthy, or untrustworthy) as a within-subjects factor and PND total score as a continuous variable. Planned Helmert contrasts of neutral vs. trustworthy and untrustworthy faces and trustworthy vs. untrustworthy faces were conducted. Results showed a significant interaction between face type and PND score *F*(2, 106) = 3.23, *p* = 0.043, η*_*p*_*^2^ = 0.06 on LPP.^3^ Planned Helmert contrasts showed no interaction of PND with face type when comparing LPP to neutral vs. trustworthy/untrustworthy faces, *F*(1, 53) = 1.34, *p* = 0.252, η*_*p*_*^2^ = 0.03, but there was a significant interaction of PND on LPP when comparing trustworthy and untrustworthy faces *F*(1, 53) = 5.60, *p* = 0.022, η*_*p*_*^2^ = 0.10 (see Figures 1A,B). In order to determine whether the effect on LPP is specific to PND, rather than poverty more generally, we ran the same analysis controlling for household income. Including household income as a covariate in the above model did not affect the statistical significance of primary results, *F*(2, 104) = 3.10, *p* = 0.049, η*_*p*_*^2^ = 0.06. Thus, the interaction between PND and trustworthy vs. untrustworthy faces revealed that higher PND, independent of household income, is related to decreased differentiation in motivated neural processing of trustworthy and untrustworthy faces.

### Neighborhood Disorder, Neural Processing of Faces, and Engagement in Criminal Behavior

**FIGURE 2:**
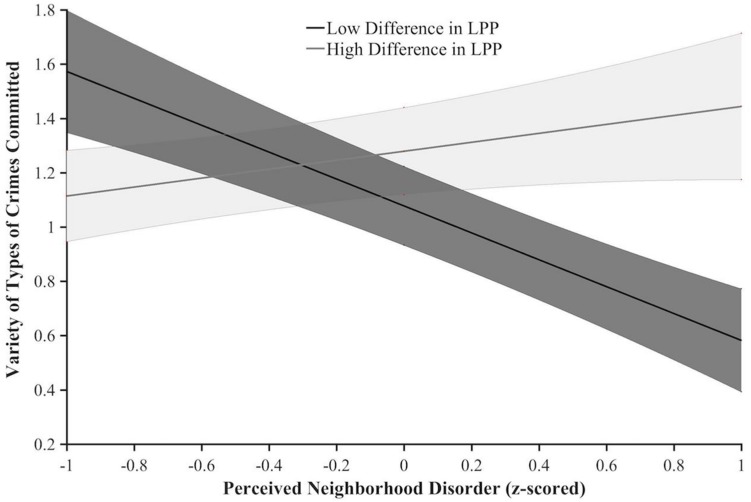
Variety of types of crime committed as a function of difference in LPP amplitude to untrustworthy and trustworthy faces (z-scored; lines represent scores at ±1 SD from the mean) and perceived neighborhood disorder (PND total z-scored). Error bands indicate 1 SE.

To investigate whether difference in neural processing of trustworthy and untrustworthy faces moderates the relationship between PND and variety of criminal engagement, a regression model was constructed as follows: X = PND total (z-scored), Y = variety in types of committed crimes (0–2), M = LPP to untrustworthy faces minus LPP to trustworthy faces (z-scored). The overall model was significant *R*^2^ = 0.18, *F*(3, 51) = 3.62, *p* = 0.019, as was the interaction term between PND and difference in LPP to untrustworthy and trustworthy faces *b* = 0.33, *t*(51) = 2.89, *p* = 0.006, which accounted for a significant proportion of variance Δ*R*^2^ = 0.14, *F*(1, 51) = 8.35, *p* = 0.006. Moreover, to examine the conditional effect of PND on engagement in crime at each level of difference in LPP to untrustworthy and trustworthy faces, this interaction was decomposed using the Johnson-Neyman procedure. At low LPP untrustworthy and trustworthy differentiation (specifically, below −0.23), PND was significantly negatively correlated with variety in types of committed crimes (*p-*value range: 0.002–0.050); however, as the difference in LPP to untrustworthy and trustworthy faces increased (i.e., >2.02), PND was significantly positively correlated with variety in types of crimes committed (*p-*value range: 0.051–0.040) (see Figure 2).

## Discussion

The impact of trust on the association between neighborhood disorder and social problems is of considerable interest and has generated mounting empirical support (Sampson, 2008; Sampson and Graif, 2009; Freedman and Woods, 2013). The present study adds to prior work by showing that individuals with higher perceptions of neighborhood disorder differ in their neural processing of facial trustworthiness. Specifically, individuals higher on perceptions of neighborhood disorder displayed less LPP differentiation between untrustworthy and trustworthy faces. Perceptions of neighborhood disorder had no effect on rating facial trustworthiness.

People are able to form trustworthiness judgments quickly from someone’s appearance with high reliability across individuals (Engell et al., 2007; Todorov et al., 2013; FeldmanHall et al., 2018). In the present study, despite variability in perceptions of neighborhood disorder, there was no difference in the ability to label a face as trustworthy or untrustworthy compared to the original ratings based on the Todorov and colleagues computer-generated faces (Oosterhof and Todorov, 2008). Even patients with prosopagnosia show normal trustworthiness judgments (Todorov and Duchaine, 2008). In fact, the only evidence, to our knowledge, that supports disruptions in the ability to provide discernable ratings for trustworthy- and untrustworthy-looking faces comes from individuals with bilateral amygdala lesions (Adolphs et al., 1998). However, while the ability to form trustworthiness judgments may be intact for various individuals, this level of assessment can be dissociated from the neural processes that subserve the formation of those judgments.

In the present study, higher perceptions of neighborhood disorder related to less differentiation in neural processing of untrustworthy and trustworthy faces, as indexed by the mean amplitude of the LPP, a neural marker of elaboration on salient stimuli. This suggests that individuals with higher perceptions of neighborhood disorder are neurally processing trustworthy and untrustworthy faces as equally salient, when previous research shows that untrustworthy faces elicit the greatest LPP response compared to trustworthy and neutral faces (Yang et al., 2011; Marzi et al., 2014; Lischke et al., 2017). One interpretation of this perceived neighborhood disorder-less LPP differentiation pattern is that it reflects increased caution engaging with salient social cues, since both positively-valenced (trustworthy faces) and negatively-valenced (untrustworthy faces) stimuli are processed similarly (Williams et al., 2006). While cautiousness is potentially adaptive in unpredictable environments (e.g., protective against potential danger, Olsson et al., 2018), an overgeneralization of that cautiousness could curtail social engagement and increase risk for some social problems in the long run (e.g., social isolation, Cacioppo and Hawkley, 2009). An alternative interpretation of this perceived neighborhood disorder-less LPP differentiation pattern is that it reflects a consequence of having less experience observing and discriminating among types of social stimuli. This interpretation stems from research that highlights increased perceptions of neighborhood disorder are associated with having fewer neighborhood social ties (Ross and Jang, 2000; Oh, 2003; Kim, 2010) and greater feelings of loneliness (Matthews et al., 2019). A separate line of research also demonstrates that decreased exposure to social experiences (e.g., institutionalized children compared to family-reared children; Moulson et al., 2009; Slopen et al., 2012) and self-reported loneliness (Cacioppo and Hawkley, 2009) are associated with abnormal patterns of neural activity to social stimuli (i.e., faces). Thus, for some individuals who perceive higher levels of neighborhood disorder, reduced differentiation in neural processing of trustworthy and untrustworthy faces could indicate experiences of social isolation or reduced exposure to social stimuli, eventually hindering opportunities to learn appropriate decoding of such stimuli.

Neighborhoods marked by disorder are spatially segregated and over-policed (Morenoff et al., 2001; Kane, 2002; Rengifo and Fowler, 2016; Logan and Oakley, 2017). This composition not only promotes a lack of trust in neighbors but also formal institutions (Steenbeek and Hipp, 2011; Nix et al., 2014; Intravia et al., 2016). Relevantly, those with higher perceived neighborhood disorder and less LPP differentiation were less likely to commit a variety of crimes, whereas those with higher perceptions of neighborhood disorder and high LPP differentiation were more likely to commit a variety of crimes. This finding further supports the interpretation that processing trustworthy and untrustworthy faces similarly may reflect a “healthy” adaptation that mitigates against deviant behavior and contacts with the law. Conversely, greater differentiation in processing of untrustworthy vs. trustworthy faces may reflect an “alertness” adaptation where ambient threat is frequently detected and results in engagement (e.g., fights) with threatening stimuli. It is important to note that while these results, taken together, support our interpretation that less differentiation in LPP amplitude to trustworthy and untrustworthy faces potentially reflects a “healthy” adaptive response to one’s environment, we are not able to determine any causality from our current study design.

Before concluding, the limitations of the present study should be noted. First, while the Todorov stimulus set is the only highly controlled and empirically validated set that manipulates the features most strongly driving trustworthiness perception, it currently contains only White faces. The majority of the present sample was Black. While race had no effect on the congruence between the present sample ratings and the computer-generated face ratings (see footnote 2), previous research suggests that race can affect social judgments (Blair et al., 2004; Stanley et al., 2011; Strachan et al., 2017) and implicit race biases can affect the extent to which an individual trusts others from different racial backgrounds (Stanley et al., 2011). Importantly, these effects related to engagement with or judgment about the behaviors of others that are perceived as untrustworthy, does not mean that there is a race-based difference in one’s ability to know what faces are signaling trustworthiness, which is what was measured in the ratings aspect of the present study. Second, though sufficient based on previous research, the sample size is small. Future research should replicate and extend this work, potentially examining variability in neural and behavioral responses based on specific social relationships or specific contexts. Third, we designed our study to investigate the LPP ERP component, given that it has been previously studied in relation to judgments of facial trustworthiness (Yang et al., 2011; Marzi et al., 2014; Lischke et al., 2017). However, we were not able to investigate how perceptions of neighborhood disorder related to early ERP components and ratings of facial trustworthiness. Future research could investigate how neural activity that reflects early attentional processes (i.e., P1) or encoding process (i.e., N170) of faces might affect trustworthiness processing and relate to individual differences in perceptions of neighborhood disorder. Further, our study design was not longitudinal, thus we were unable to establish causality amongst our variables of interest. Future research should investigate changes in ERP components to facial trustworthiness and perceptions of neighborhood disorder over time, in order to understand causal relationships between brain activity and social stimuli, and differences in social environments.

Overall, the present study provides evidence that perceptions of neighborhood disorder differentially relate to the way facial trustworthiness is processed. Moreover, these differences may be protective against social problems (i.e., criminal behavior). Exploring the association between neighborhood characteristics and variability in individual-level processing is important for refining our understanding of what shapes our beliefs and behaviors in navigating social landscapes.

## Data Availability Statement

The datasets generated for this study are available on request to the corresponding author.

## Ethics Statement

The studies involving human participants were reviewed and approved by the Yale University Human Investigation Committee. The patients/participants provided their written informed consent to participate in this study.

## Author Contributions

S-AC and AB-S designed the experiment. S-AC collected and analyzed the data under the supervision of AB-S. S-AC wrote the article with AB-S providing critical revisions.

## Conflict of Interest

The authors declare that the research was conducted in the absence of any commercial or financial relationships that could be construed as a potential conflict of interest.

## Footnotes

1 https://www.neighborhoodatlas.medicine.wisc.edu/

2 Separate binary logistic regressions were run with inclusion or exclusion due to quality of EEG data as the dependent variable and age, education level, race, IQ, household income, and PND scores as the independent variables. Participants excluded due to quality of EEG data did not differ significantly from the included participants in age, education level, race, IQ, or PND scores (*p* > 0.05). Participants that were excluded due to quality of EEG data differed significantly from the included participants on household income, such that included participants were more likely to come from households with lower household incomes Odds Ratio = 0.82, *B* = −0.20, *p* = 0.025.

3 There was no effect of race on mean LPP amplitude in a GLM with face type (neutral, trustworthy, or untrustworthy) as a within-subjects factor and race as a between-subjects factor, *F*(2, 106) = 1.09, *p* = 0.340. Moreover, race did not moderate the association between PND and face type LPP, *F*(2, 102) = 0.44, *p* = 0.645.
